# Thinking about touch facilitates tactile but not auditory processing

**DOI:** 10.1007/s00221-012-3020-0

**Published:** 2012-02-22

**Authors:** Helen A. Anema, Alyanne M. de Haan, Titia Gebuis, H. Chris Dijkerman

**Affiliations:** 1 Experimental Psychology, Helmholtz Institute, Utrecht University, Utrecht, The Netherlands; 2 Department of Neurology, University Medical Centre Utrecht, Utrecht, The Netherlands; 3 Department of Public Health, Academic Medical Centre, University of Amsterdam, Meibergdreef 9, 1100 DD Amsterdam, The Netherlands; 4 Laboratory of Experimental Psychology, University of Leuven (K.U. Leuven), Leuven, Belgium

**Keywords:** Tactile, Somatosensory, Tactile imagery, Perception, Human

## Abstract

Mental imagery is considered to be important for normal conscious experience. It is most frequently investigated in the visual, auditory and motor domain (imagination of movement), while the studies on tactile imagery (imagination of touch) are scarce. The current study investigated the effect of tactile and auditory imagery on the left/right discriminations of tactile and auditory stimuli. In line with our hypothesis, we observed that after tactile imagery, tactile stimuli were responded to faster as compared to auditory stimuli and vice versa. On average, tactile stimuli were responded to faster as compared to auditory stimuli, and stimuli in the imagery condition were on average responded to slower as compared to baseline performance (left/right discrimination without imagery assignment). The former is probably due to the spatial and somatotopic proximity of the fingers receiving the taps and the thumbs performing the response (button press), the latter to a dual task cost. Together, these results provide the first evidence of a behavioural effect of a tactile imagery assignment on the perception of real tactile stimuli.

**Electronic supplementary material:**

The online version of this article (doi:10.1007/s00221-012-3020-0) contains supplementary material, which is available to authorized users.

## Introduction

To many of us, it is easy to imagine how a creepy spider crawls down our back or how it feels like when grasping a handful of rice. It is currently widely accepted that mental imagery and real perceptual processes overlap substantially. However, the extent to which imagery and real sensory representations are shared is debated. Contrary to perceptual processes, the primary sensory cortices are not always activated during imagery (i.e. Pylyshin 2002; Amedi et al. 2008). Mental imagery is most frequently investigated in the visual, auditory and motor domain (imagination of movement) with the investigation of tactile imagery (imagination of touch) being relatively neglected. Studies that do investigate tactile imagery, however, focus on the similarity between neural mechanisms underlying tactile imagery and real touch (Davidson and Schwartz 1977; Uhl et al. 1994; Fallgatter et al. 1997; Yoo et al. 2003; Olivetti Belardinelli et al. 2009). Within the field of visual and auditory imagery research, both the underlying neural networks (see for overview Kosslyn et al. 2001 and Pylyshin 2002) as well as imagery effects on behaviour have been investigated frequently (e.g. Ishai and Sagi 1997; Craver-Lemley and Arterberry 2001; Pearson et al. 2008). To our knowledge, however, behavioural effects of tactile imagery on the processing of ‘real’ tactile stimuli have not yet been investigated.

In line with the theory that visual and auditory imagery share neural networks with perceptions of physical stimuli, tactile imagery seems to have at least partial overlap with neural mechanisms underlying real perception (Davidson and Schwartz 1977; Uhl et al. 1994; Fallgatter et al. 1997; Yoo et al. 2003). Davidson and Schwartz (1977), Fallgatter et al. (1997) and Uhl et al. (1994) observed that the parietal cortex was activated to a larger extent than the occipital cortex during tactile imagery. Yoo et al. (2003) further elaborated on this finding and observed tactile imagery-related activation in the contralateral primary and secondary somatosensory cortices, the left parietal lobe, left inferior frontal gyri, left dorsolateral prefrontal area, left precentral gyrus, left insula and medial frontal gyrus.

The way imagery is induced and the kind of stimuli that have to be imagined in these studies vary considerably, suggesting that the cortical activation that tactile imagery evokes is not restricted to a certain combination of stimulus and/or imagery instruction. For instance, imagery content was induced on the basis of both short-term memory, a tactile stimulus presented just prior to the imagery assignment (Davidson and Schwartz 1977; Uhl et al. 1994; Yoo et al. 2003), and long-term memory, traces activated by verbal instructions (Fallgatter et al. 1997). The type of stimuli that were used for imagination varied between simple vibration sensations (Davidson and Schwartz 1977), a gentle stroke of a Von Frey Hair (Yoo et al. 2003) and less simple stimuli such as textures (Uhl et al. 1994). Thus, tactile imagery evoked by a variety of induction methods induces activation of neural processes associated with processing of actual somatosensory stimuli.

The fact that imagery and perception share neural circuits suggest that these processes can affect one another. Behavioural studies, investigating the effects of visual and auditory imagery on perception of stimuli, showed that the timing and the content of imagery instruction determines whether facilitation or inhibition of conscious processing is observed (Segal and Fusella 1970; Farah and Smith 1983; Ishai and Sagi 1997; Craver-Lemley and Arterberry 2001; Pearson et al. 2008). Segal and Fusella (1970) investigated how detection of a visual (blue arrow) or auditory signal (harmonica chord) was affected by visual and auditory imagery. They presented the visual or auditory signal exactly at the time participants indicated they had a clear image for 2 s. The results revealed that detection of the signal was poorer for the imagery condition than the no-imagery condition, and detection of the signal was even poorer when signal and imagery came from the same modality. It was concluded that presentation of the real stimulus during imagery results in a confusion of the imagined and the real stimulus. Facilitatory effects of visual imagery on the perception of physical stimuli can be induced as well. Farah and Smith (1983), for instance, revealed that imagining an auditory stimulus *before* the presentation of a physical auditory stimulus (tone) produces lower detection thresholds as compared to imaging *during* the presentation of the tone. This effect was stronger for the condition in which frequencies of imagery and stimulus were similar as compared to imagining a different frequency. Facilitatory or inhibitory effects not only depend on the timing but also on the feature similarity between the physical and imagined stimulus and the task difficulty (Finke 1986). More specifically, facilitatory processes are more likely to arise when the physical and imagined stimulus are more resembling, while inhibitory processes are more likely to arise with increasing task difficulty. Together, these studies suggest that different factors play an important role in the size and direction of the effect imagery has on the processing of real stimuli.

In all, the above-mentioned studies provide substantial evidence that imagery and perception share representations. In the visual and auditory domain, the overlap is reflected in perceptual behaviour. That is, imagery and perception may facilitate or inhibit each other, depending on the temporal interval between the perceived and imagined stimulus. In the tactile domain, such behavioural influences of imagery on perception have not been investigated yet. The current study therefore sought to demonstrate that tactile imagery can affect the processing of real tactile stimuli. If imagery and real perception share common neural substrates, one could expect that the activation of tactile higher order knowledge derived during (imagined) common haptic activities will influence the perception of real physical stimuli. To investigate exactly this, we used a spatial left/right discrimination task of tactile and auditory stimuli after a tactile or auditory imagery assignment was given. Specific imagery content was determined by visual stimuli informative of both tactile and auditory sensations (e.g. gravel and marbles). We expected that tactile imagery would facilitate conscious processing of tactile real stimuli relative to auditory stimuli. Similarly, we expected that auditory imagery would facilitate conscious processing of auditory real stimuli relative to tactile stimuli.

## Materials and methods

### Participants

Fifteen healthy participants of Utrecht University (7 men, 8 women, mean age 24.8 ± 3 years, two left-handed) participated in this study. All participants, who signed an informed consent form, were naive to the purpose of the experiment and received either a small payment or course credits required as part of their studies. This study was approved by the local ethical medical board and has been conducted in accordance with the Declaration of Helsinki.

### Stimuli and set-up

**Fig. 1:**
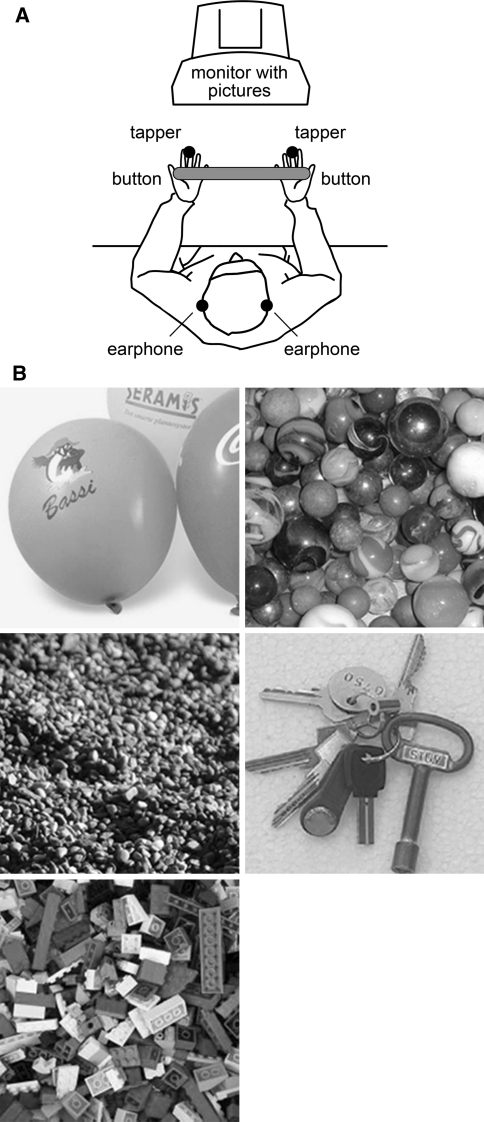
**a** Experimental set up. **b** Stimuli used to generate imagery (color figure online)

Participants were seated at a table in front of a monitor with their hands in parallel (±30 cm apart) on top of the table. The hands (palm up) were placed underneath a response device, so that the thumbs of each hand could press a button (Fig. 1a).

Tactile stimulation (further referred to as tap) was delivered by a 2-mm-thick metallic rod propelled by a computer-controlled miniature solenoid with a duration of 5 ms (MSTC3 M&E Solve, Rochester, UK: http://www.me-solve.co.uk). The skin indentation evoked by the solenoid produced a well above detection threshold stimulus. Solenoids were attached to the dorsal (distal) part of the left and right little, ring, middle and index finger. To prevent for skin irritation induced by the solenoid, the exact stimulus location was altered between different blocks of trials. Auditory stimuli consisted of 20 ms pink noise (further referred to as beep) and were presented into the left or right ear using E-A-RTONE 3A insert earphones (Earlink Inc.).

The pictures used to guide imagery in our study consisted of pictures informative of tactile as well as auditory sensations. The selection of the imagery stimuli was based on two separate pilot studies. First, we selected 10 pictures that were associated with touch and sound and with only few other associations out of a total of 131 (see online supplementary material for more information about the imagery stimuli). To this end, 48 participants were presented (beamer in lecture hall) with 131 pictures (2 trials per picture) that were collected on the Internet. Participants were required to judge what type of association they had with the picture: taste, smell, touch or sound. We then selected ten pictures that were both rated as associated with touch and sound, and with only few other associations (e.g. a key chain has strong tactile associations AND makes a sound). Next, in a second pilot experiment, we tested whether it was possible to generate both tactile and auditory imagery sensations on the basis of the pictures content by asking participants (*N* = 7) to press a button when a clear imagined sensation was obtained. Pictures were presented on a computer screen. Each picture was presented six times. The specific instructions were as follows: ‘imagine you hear the sound the content of the picture evokes’ (auditory), or ‘imagine you feel the sensation to the skin of your dominant hand and fingers that the content of the picture evokes’. Using pictures of which its content can be used to evoke tactile and auditory imagery allowed us to use keep the visual input equal in both the auditory and the tactile imagery conditions. To obtain insight about the duration of the imagery, it was additionally asked to keep the imagined (auditory or tactile) sensation active as long as possible. Participants had to press the button again when they ‘lost’ the imagined sensation, after which a new trial started. When participants were not able to generate imagery and no response was given during the entire trial, the trial ended automatically after 10 s. After the experiment, participants were required to judge the ten pictures on the basis of the vividness of the imagery they evoked. Overall, participants responded to have a clear mental image in 2,361 ms (standard error mean SEM = 447) and rated the vividness on average seven out of 10 (standard deviation SD = 0.6). In the auditory imagery condition, participants responded in 2,346 ms (SEM = 337) and scored on average 6.7 (SD = 1.3). Almost all participants [95% (SD 1%)] were able to maintain an active imagined sensation for at least 5 s. Five pictures were selected on the basis of three criteria: (1) their vividness score (score >7), (2) a less than 1.5-point difference between the tactile and auditory score and (3) the time needed to generate an imagined sensation (RT <2,250 ms; see for selected imagery pictures Fig. 1b).

### General procedure

The study consisted of three consecutive parts: (1) a baseline block of trials testing the left/right discrimination of tactile and auditory stimuli without imagery, (2) an imagery practice task and (3) the imagery block of trials that investigated left/right discrimination of tactile and auditory stimuli after mental imagery. In short, the imagery experiment consisted of a left/right discrimination task which required participants to first imagine a certain sensation (touch or sound), indicated by a picture presented on a computer screen, and subsequently had to decide whether a real touch or sound was delivered on the left/right ear or hand. Below, we describe the procedures of the separate parts of the study in chronological order.

### Procedure baseline block

To get insight in a possible difference in reaction time to the beeps or taps, we first ran a baseline block of trials. This experiment was almost identical to the main experiment except that subjects, who were naïve to the topic of the study, only had to attend the visual scene presented. They were not instructed to perform any imagery of the related sensation (touch or sound).

A trial lasted for 5,500 ms and started with the presentation of one of the five imagery pictures that participants were instructed to view attentively. The picture would remain on the computer screen for 5,500 ms. This time interval was chosen on basis of the second pilot experiment, which showed that most participants were able, after practicing, to generate a mental image (average = 1,419 ms; SD = 252 ms). Almost all participants (95%; SD = 1%) were able to keep the imagined sensation (auditory or tactile) active for at least 5 s. So, after attentively viewing the picture for 2,500 ms, either a tap or beep was delivered to the left or right ear or finger. To prevent anticipation towards these stimuli, taps and beeps were delivered randomly within a 3-s time interval. Participations were required to give a speeded left/right response by pressing with the left thumb on the left button for left-sided stimuli and vice versa.

In total, 60 trials were presented, 12 trials per imagery picture (5 pictures in total), of which 30 beeps and 30 taps, equally delivered to the left and right. Imagery pictures and taps and beeps were presented randomly. An additional 10 practice trials were performed prior to the task. After the baseline measurement, participants were informed about the imagery task ahead.

### Procedure imagery practice task

To train subjects in creating vivid and stable images of a specific tactile or auditory sensation in short notice, we presented the subjects with a training session. This was done prior to the imagery block of trials to keep the participants naive to the imagery content of the study.

Within two separate blocks (auditory and tactile; 30 trials in total), participants were instructed to imagine the sensation to the hands and fingers or the sound sensation provoked by the presented image on the computer screen. Specifically, it was asked: ‘imagine you hear the sound the content of the picture evokes’ (auditory), or ‘imagine you feel the sensation to the skin of your dominant hand and fingers that the content of the picture evokes’. It was encouraged to keep the imagined (auditory or tactile) sensation active as long as possible. When a vivid experience of the requested sensation was obtained, participants had to press a button. The picture remained on the computer screen for an additional 5,000 ms (10,000 ms in total) during which participants were instructed to maintain the imagined sensation. When participants ‘lost’ the imagined sensation, they had to press the button after which a new scene appeared. Participants finished the imagery practice by rating each visual scene on the amount of effort it took to generate the required sensation using a 10-point Likert scale (0 = no imagery at all, 10 = extremely easy to generate image). Next, the main experiment started.

### Procedure imagery block

The main imagery experiment was the same as the baseline measurement except that subjects were now instructed to attentively focus on the visual scenes to generate the required auditory or tactile sensation, using the same imagery instruction scripts as were given in the imagery practice task.

The type of imagery was blocked, whereas the beeps and taps were presented in a random order within each block of trials. Participants completed a total number of six blocks (3 touch and 3 auditory imagery), and the order of the blocks was counterbalanced across the participants. Each imagery block consisted of 20 trials, 4 trials per visual scene, two taps and two beeps, presented equally often to the left and the right side. Together, each imagery × stimulus condition was presented 30 times (6 per visual scene).

In sum, the effect of tactile and auditory imagery on the discrimination of left and right beeps and taps was measured using a 2 [Imagery type: Auditory imagery (AI), Tactile imagery (TI)] × 2 (Stimulus Type: Beep, Tap) factorial within-subjects design with reaction time, time between the onset of the beep or tap and the response, as dependent variable. All factors and their subsequent levels were counterbalanced in the experimental procedure.

### Data analyses

First, for the imagery practise task, the average time needed to generate an image was calculated for each visual scene. Participants who could be identified as outliers were eliminated for further analyses.

Second, for the imagery experiment [Baseline (BL) and Imagery block of trials] separate reaction times of correct responses for beeps and taps were calculated for each task (BL, AI, TI) and stimulus type (Beep, Tap) and cleaned from outliers (±2.5 SD). Analyses were performed on the reaction times for stimuli presented between 3 and 5 s after imagery onset, since this interval was thought to represent the interval in which performance was the least biased by double task costs (‘early’ trials 2.5–3 s after imagery onset) and by anticipation of ‘late’ trials (5–5.5 s after onset). Differences in baseline reactions to beeps and taps (BL) were investigated using a paired sample *t* test. Reaction times of the two imagery conditions were tested using a 2 (Imagery type) × 2 (Stimulus Type) repeated measures ANOVA (GLM) with reaction time as independent variable. Significant interactions were further explored using paired samples *t* tests.

## Results

### Imagery practise task

On average, in 1% (SD = 0.04%) of the trials, participants were unable to generate an image, and in 5% of the trials, an imaged sensation could not be maintained. Data of these trials were discarded for the analysis of reaction times. Overall, participants needed on average 1,873 ms to generate a vivid image (SD = 647 ms; min = 681 ms, max = 7,319 ms), and there was no clear difference between auditory and tactile imagery reaction times (auditory = 1,975 ms (SD = 600 ms); tactile = 1,753 ms (SD = 694). One participant (mean = 676 ms, SD = 733 ms) was excluded from the study on the basis of an overall low amount of time needed to generate a tactile image and remarkable variation in reaction times. The rates participants gave to the imagery pictures (0 = no imagery at all, 10 = extremely easy to generate image) were overall positive. The auditory ratings were on average 6.8 (SD = 1.2), whereas the tactile ratings scored significantly higher [(mean = 7.7 SD = 0.8; *t* (13) = −2.511, *p* < 0.05)].

### Imagery experiment

Participants performed almost perfect on the left/right discrimination task as on average only 0.9% (SE = 0.5%) of the trials were incorrect. In total, 112 trials were discarded (2.3%), of which 168 trials on the basis of outliers (1.8%) and 44 on the basis of incorrect trials (0.5%) equally divided over the imagery conditions (AI: outlier = 53, incorrect = 14; AI: outlier = 45, incorrect = 14), but with more outliers in the baseline (outlier = 70) as compared to the imagery condition.

**Fig. 2:**
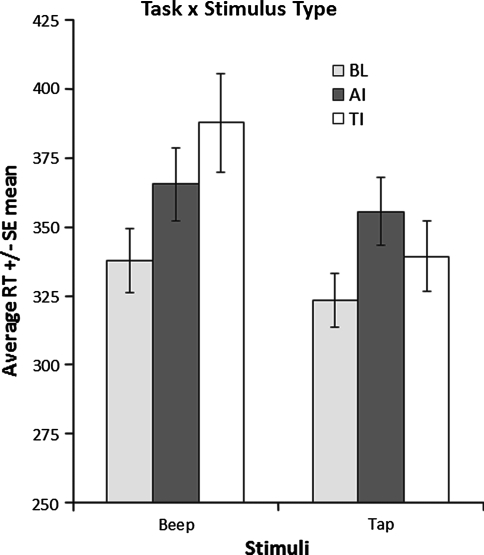
Mean reaction times of left/right discriminations of taps and beeps for each task × stimulus type condition. *BL* baseline, *AI* auditory imagery, *TI* tactile imagery. *Note*: reaction times in milliseconds, *error bars* are between subjects standard errors around the mean (color figure online)

In the baseline condition, participants responded faster, but not significantly, to taps (332 ms ± 11) as compared to beeps (338 ms ± 9). The 2 × 2 ANOVA revealed a significant main effect of stimulus type. Taps were responded to faster (347 ms ± 12) as compared to beeps (376 ms ± 15; *F* (1,13) = 10.40, *p* < 0.01). As was hypothesized, task interacted significantly with stimulus type (*F* (1,13) = 24.99, *p* < 0.01; see Fig. 2). Taps were responded to significantly faster after a tactile imagery assignment (339 ms ± 13) as compared to an auditory imagery assignment (355 ms ± 12; *t*(13) = 2.330, *p* < 0.05). Also, beeps were responded to significantly faster after an auditory imagery assignment (365 ms ± 13) as compared to a tactile imagery assignment (388 ms ± 18; *t*(13) = 3.236, *p* < 0.01). Together, these results suggest that imagery relatively facilitates the reaction towards modality congruent real stimuli.

To gain more insight into the effects that our imagery stimuli generated, we tested whether pictures with higher vividness scores were easier (faster) to generate a mental image. Therefore, we correlated the vividness scores per picture that participants assigned after the entire experiment to the average reaction times (‘yes imagery’) per picture obtained in the imagery practice task. It has to be noted, however, that the power of the correlation test is limited as we only had five data points per participant and only 15 participants. The results revealed that the auditory vividness score of picture 1 and 2 correlated negatively (one-sided Bonferroni corrected) with the time a participant needed to generate the required image (Pearson’s *R* = −0.73; *p* < 0.01 and *R* = −0.68; *p* < 0.01, respectively). Also, the tactile vividness score of picture 2 correlated negatively with the time participants needed to generate the required image (Pearson’s *R* = −0.54; *p* < 0.02). All other correlations were, although negatively associated, not substantial enough to reach significance.

## Discussion

The current study investigated the effect of tactile and auditory imagery on the left/right discriminations of tactile and auditory stimuli. In line with our expectations, tactile imagery resulted in faster discrimination of tactile than auditory stimuli and vice versa. Overall, tactile stimuli were responded to faster as compared to auditory stimuli, and stimuli in the imagery condition were on average responded to slower as compared to baseline performance (left/right discrimination without imagery assignment). Together, these results provide the first evidence of a behavioural effect of tactile imagery on the perception of real tactile stimuli.

Besides the main finding of relative same modality facilitation, the results also revealed that participants responded overall slower to stimuli in the imagery condition compared to baseline (no imagery). A dual task cost of left/right discrimination performance and mental imagery most likely explains this result. Also, tactile stimuli were responded to faster as compared to auditory stimuli. Participants had to indicate their responses for both the somatosensory and auditory discrimination with their thumbs. The smaller spatial and somatotopic proximity between the fingers receiving the somatic stimulus and the thumbs used to respond as compared to the auditory condition most likely explains this difference in response time across conditions.

Overall, we observed less interference of the imagery task when imagery and the physical stimulus were from the same modality. On the assumption that tactile imagery and tactile perception share neural substrates, one could argue that when our participants imagined a tactile sensation (e.g. the tactile sensation of grasping a hand full of gravel), somatosensory areas were activated, perhaps via modality-specific attentional processes. Indeed, attention to tactile information modulates activity in both the primary and secondary somatosensory areas (Johansen-Berg et al. 2000).

Switching attention between two modalities induces a ‘modality switch cost effect’, explaining the slower reaction times in the between-modality condition as compared to the within-modality condition. In the former case, participants had to direct their attention away from the imagined tactile sensation in order to attentively process the auditory stimulus and vice versa. Behavioural evidence for such modality switching costs in the perception of multiple ‘real’ (tactile) stimuli was demonstrated by Turatto et al. (2004; see also Spence et al. 2001). The authors investigated cross-modality attentional shifts between, for example, the auditory and the tactile modality by applying two successive stimuli, a tactile stimulus or a sound, and having participants judge whether the middle or index finger was stimulated or whether the sound was high or low pitch. It was observed that intramodal stimuli were responded to faster as compared to cross-modal stimuli, even when participants had the opportunity to focus on the target modality and were able to prevent involuntary ipsimodal capture of attention.

So far, it can be suggested that our response pattern resembles that of the attentional modality switch effect. We, however, studied the effect of a relatively *complex* imagined stimulus on the perception of a relatively *simple* lower-order stimulus, while previous studies used a relatively *simple* stimulus for both the real and imagined stimulus (see for overview of the tactile literature: Spence and Gallace 2007). Modality switch effects between the processing of simple and complex stimuli have been observed by Van Dantzig et al. (2008). The authors investigated whether perceptual tasks and conceptual tasks (such as mentally verifying whether a lemon is yellow) are grounded within the same system. According to the perceptual symbols theory, to represent the concept of for example ‘chair’, visual, tactile, motor and emotional networks are activated to re-enact the experience of the chair. To assess whether these conceptual representations were based on perceptual processing systems, the authors investigated the effect of a perceptual task (e.g. left/right discrimination of a tactile vibration) on a subsequently performed conceptual task (e.g. whether a property was true for a specific concept ‘banana is yellow’), both across and within modality. The results revealed that the responses on the conceptual task were slower when preceded by a different modality perceptual task. This underlines the hypothesis that perceptual and conceptual representations are partially based on the same systems. In addition, it might explain why we observed attentional effects of an imagery task encompassing conceptual information, on a simple perceptual left/right discrimination task.

One aspect of our study requires some discussion. It has to be noted that we cannot entirely rule out the possibility that participants used both visual and kinaesthetic imagery (somatosensory sensations of movement) together with the imagined tactile sensations such as roughness, edges, etc., when imagining the tactile qualities of the presented scene. By asking the participants to attend to the tactile sensation of the target object to the skin of the hands and fingers, we aimed to emphasize the tactile more than the haptic quality of perception. As haptic experience with our environment is multimodal in essence, it can be assumed, however, that automatically other sensations are mentally activated but to a lesser extent.

The multimodal aspect of the haptic sense provides some potentially interesting clinical implications. Kinaesthetic/motor imagery (mental imagery of movement) is frequently used as training paradigm in motor (re) learning in sports (Murphy 1994) and in neurologically impaired populations (Dijkerman et al. 2004; Liu et al. 2004; Page et al. 2007). Mentally simulating the execution of a movement activates the motor network (see for review De Lange et al. 2008). When practised frequently, this can lead to improvements of motor function. Although elaboration on its mechanisms is beyond the scope of this article, engaging in tactile imagery while training upper limb hand function might be an important factor for motor imagery training to be effective. Although small-sized clinical trials have shown positive effects of training, these effects have not been convincing so far (Zimmermann-Schlatter et al. 2008). It could be hypothesized that adding tactile imagery to the commonly used visually oriented motor imagery training paradigm might yield a larger training effect. In everyday life, tactile and motor function of the upper limbs is tightly connected, which is embedded in the anatomy of the somatosensory system (Dijkerman and De Haan 2007). Including tactile imagery in motor imagery training might therefore drive motor learning and recovery more effectively.

Together, we observed that tactile imagery facilitated left/right discrimination of tactile stimuli relative to auditory stimuli, and auditory imagery relatively facilitated the left/right discrimination of auditory stimuli. Furthermore, we demonstrated that imagery of ‘higher-order’ conceptual information affected the processing of ‘lower-order’ less complex perceptual information. This is consistent with the idea that top-down expectations or recollections of previous (tactile) experiences shape (tactile) perception itself (Pearson et al. 2008).

## Electronic supplementary material

Below is the link to the electronic supplementary material.

Supplementary material 1 (DOCX 13 kb)

Supplementary material 2 (PSD 21241 kb)

Supplementary material 3 (PSD 22952 kb)

Supplementary material 4 (PSD 26100 kb)

Supplementary material 5 (PSD 10504 kb)
