# Supplementary material for: Thinking about touch facilitates tactile but not auditory processing
Source: Exp Brain Res. 2012 Feb 22;218(3):373–80. doi: 10.1007/s00221-012-3020-0 (PMC3324683; doi:10.1007/s00221-012-3020-0)
Supplement: Supplementary file 1 — Supplementary material 1 (DOCX 13 kb) [file 221_2012_3020_MOESM1_ESM.docx]

The pictures used to guide imagery in our study consisted of pictures informative of tactile as well as auditory sensations. The selection of the imagery stimuli was based on two separate pilot studies. First, we selected 10 pictures that were associated with touch and sound and with only few other associations out of a total of 131. In order to do so, 48 participants were presented (beamer in lecture hall) with 131 pictures that were collected on the internet and adapted to fit the pilot experiment. Participants were required to judge what type of association they had with the picture: taste, scent, touch, or sound. We then selected 10 pictures that were both rated as associated with touch and sound, and with only few other associations (e.g. a key chain has strong tactile associations AND makes a sound). Next, in a second pilot experiment we tested whether it was possible to generate both tactile and auditory imagery sensations on basis of the pictures content. From this second pilot study we selected the 5 final pictures (stimuli tagged with Exp).
